# Caspase-2 promotes obesity, the metabolic syndrome and nonalcoholic fatty liver disease

**DOI:** 10.1038/cddis.2016.19

**Published:** 2016-02-18

**Authors:** M V Machado, G A Michelotti, M L Jewell, T A Pereira, G Xie, R T Premont, A M Diehl

**Affiliations:** 1 Division of Gastroenterology, Department of Medicine, Duke University Medical Center, Durham, NC, USA; 2 Gastroenterology Department, Hospital de Santa Maria, Lisbon, Portugal

## Abstract

Obesity and its resulting metabolic disturbances are major health threats. In response to energy surplus, overtaxed adipocytes release fatty acids and pro-inflammatory factors into the circulation, promoting organ fat accumulation (including nonalcoholic fatty liver disease), insulin resistance and the metabolic syndrome. Recently, caspase-2 was linked to lipoapoptosis, so we hypothesized that caspase-2 might be a critical determinant of metabolic syndrome pathogenesis. Caspase-2-deficient and wild-type mice were fed a Western diet (high-fat diet, enriched with saturated fatty acids and 0.2% cholesterol, supplemented with fructose and glucose in the drinking water) for 16 weeks. Metabolic and hepatic outcomes were evaluated. *In vitro* studies assessed the role of caspase-2 in adipose tissue proliferative properties and susceptibility for lipoapoptosis. Caspase-2-deficient mice fed a Western diet were protected from abdominal fat deposition, diabetes mellitus, dyslipidemia and hepatic steatosis. Adipose tissue in caspase-2-deficient mice was more proliferative, upregulated mitochondrial uncoupling proteins consistent with browning, and was resistant to cell hypertrophy and cell death. The liver was protected from steatohepatitis through a decrease in circulating fatty acids and more efficient hepatic fat metabolism, and from fibrosis as a consequence of reduced fibrogenic stimuli from fewer lipotoxic hepatocytes. Caspase-2 deficiency protected mice from diet-induced obesity, metabolic syndrome and nonalcoholic fatty liver disease. Further studies are necessary to assess caspase-2 as a therapeutic target for those conditions.

Western societies exist in an era of caloric excess that is at odds with evolutionary adaptations to the ancestral low-calorie lifestyle. As Western dietary habits have spread, obesity prevalence has nearly doubled worldwide. In 2008, 1.4 billion adults were overweight and half a billion obese, comprising 35 and 11% of the world population.^1^ The metabolic syndrome is a cluster of dysfunctions including abdominal obesity, hypertriglyceridemia, low high-density lipoprotein-cholesterol levels, hypertension, and glucose intolerance/insulin resistance/type 2 diabetes mellitus (T2DM).^2^ Obesity increases the risk for all aspects of metabolic syndrome, related cardiovascular disease and multiple cancers. Ectopic fat accumulation in the liver, dubbed nonalcoholic fatty liver disease (NAFLD), strongly associates with the metabolic syndrome.^3, 4^ Whereas metabolic disturbances, including insulin resistance undoubtedly can induce hepatic steatosis, whether or not hepatic steatosis can induce insulin resistance is still a matter of debate. On one hand, epidemiological studies suggest that patients with NAFLD are at increased risk for developing T2DM.^5^ On the other, several mouse models of NAFLD do not develop insulin resistance, and even exhibit increased sensitivity to insulin.^6, 7, 8^

Adipocytes respond maladaptively to chronic energy surplus, resulting in adipocyte hypertrophy.^9^ Fat-swollen adipocytes become insulin resistant, decrease expression of adipokines such as the anti-inflammatory insulin-sensitizer adiponectin, and increase expression of the satiety factor leptin.^10^ Stressed adipocytes undergo cell death in mice with genetic- or diet-induced obesity, and in morbidly obese humans.^9, 11^ Adipocyte cell size and cell death correlate with the presence of metabolic syndrome, insulin resistance and NAFLD.^9, 10, 12^

Caspase-2 is an initiator caspase for cellular apoptosis.^13^ The primary function of caspase-2 remains to be determined, however, because it has also been implicated in several other vital processes including regulation of cell cycle checkpoints, oxidative stress responses, autophagy and senescence.^14, 15, 16, 17^ Intriguingly, caspase-2 mRNA expression is elevated in adipose tissue of rats fed high-fat diet.^18^ Recently, caspase-2-initiated apoptosis was associated with lipotoxicity caused by accumulation of saturated fatty acids in frog oocytes,^19^ cultured mouse hepatocytes, and in mouse and human NAFLD.^20^ Caspase-2 expression strongly correlated with liver disease severity in patients with NAFLD.^20^ Further, caspase-2 depletion protected mice with methionine/choline-deficient (MCD) diet-induced steatohepatitis from hepatocyte apoptosis and fibrosis progression.^20^

We hypothesized that caspase-2 may be a key link between energy surplus, adipocyte death and the development of the metabolic syndrome and NAFLD. Our data support this concept because caspase-2-deficient mice fed a Western diet were protected from many aspects of the metabolic syndrome, namely increased adiposity, insulin resistance, dyslipidemia and NAFLD development and progression. These results suggest caspase-2 as a target for managing the metabolic syndrome, obesity, T2DM and NAFLD.

## Results

### Caspase-2-deficient mice are protected from diet-induced obesity

**Figure 1:**
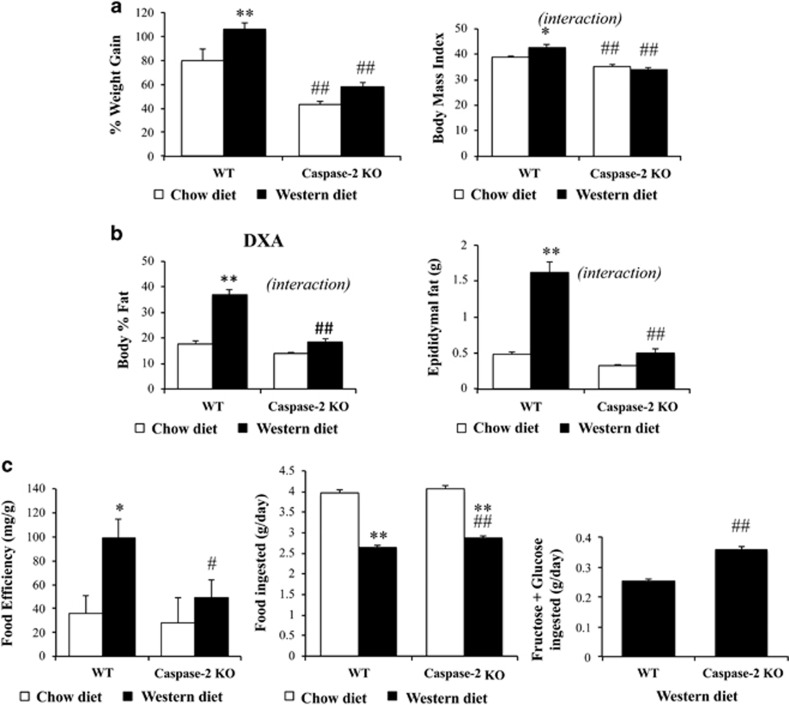
Caspase-2-deficient mice are protected from diet-induced obesity. (**a**) Right panel: weight increase in caspase-2 knockout and WT mice-fed chow *versus* Western diet (four animals per genotype in chow diet groups and eight animals per genotype in Western diet groups), at the end of treatment as compared with baseline measurements. Left panel: body mass weight. (**b**) Body mass % fat (DXA) and epididymal fat weight. (**c**) Food efficiency (weeks 2–15 of treatment); food and high-corn syrup equivalent ingested during treatment. The errors reported represent mean±S.E.M. *P<0.05, **P<0.01 chow *versus* Western diet; ^#^*P*<0.05, ^##^*P*<0.01 WT *versus* knockout mice. Interaction, assessed by two-way ANOVA, indicates that the effect of diet was different between genotypes

Caspase-2-deficient mice and wild-type (WT) controls were fed standard chow diet or high-fat, high-sugar Western diet for 16 weeks. At the end of treatment, WT mice fed Western diet weighed 10% more than WT mice-fed chow diet. However, Western diet induced minimal additional weight gain in caspase-2-deficient mice (Figure 1a; Supplementary Figure 1). Similar differences were seen in the body mass index (Figure 1a). Interestingly, the decreased weight in caspase-2 KO mice was not related to impaired growth, as caspase-2 KO mice were longer than WT mice by the end of the experiment (Supplementary Figure 1). As expected, Western diet doubled body fat in WT mice (assessed by dual-energy X-ray absorptiometry (DXA)) compared with chow diet, and tripled abdominal adipose tissue. Western diet-induced adiposity was strikingly blunted in caspase-2-deficient mice (Figure 1b). This is noteworthy since, more than obesity itself, abdominal *versus* subcutaneous distribution of body fat is deleterious.^21^

Differences in adiposity could not be explained by decreased food intake, as caspase-2-deficient mice ate 8% more solid food and drank 40% more sugar-supplemented water than WT mice. Indeed, WT mice had higher food efficiency, gaining more weight per gram of food ingested (Figure 1c).

### Caspase-2-deficient mice are protected from diet-induced impairments in glucose metabolism

**Figure 2:**
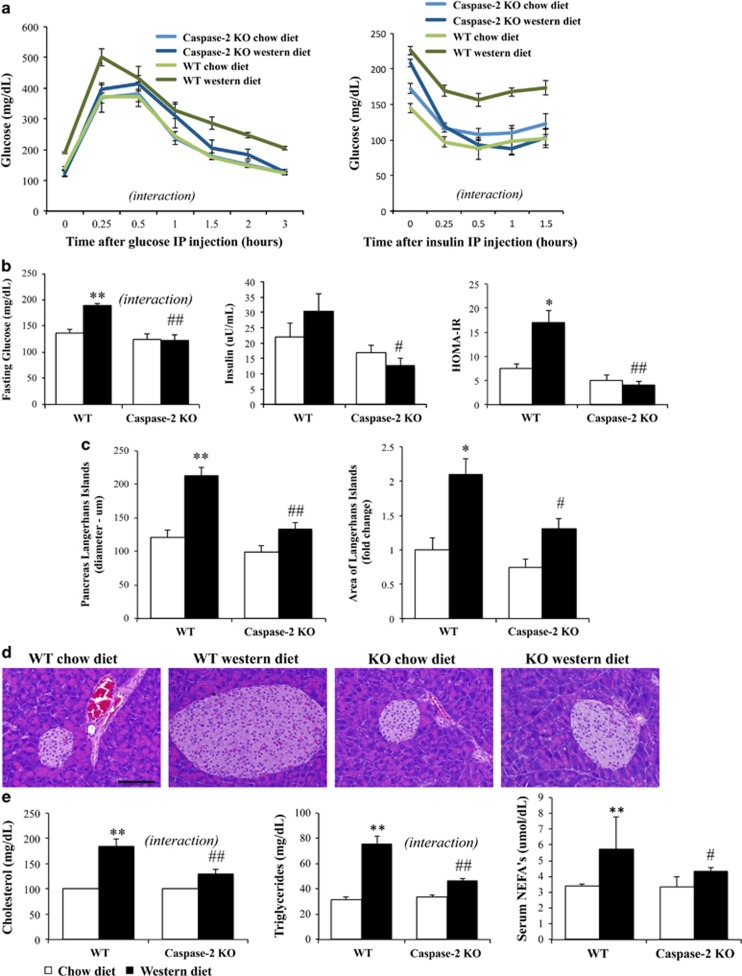
Caspase-2-deficient mice are protected from diet-induced diabetes mellitus and dyslipidemia. (**a**) Right panel: glucose tolerance test after 15 weeks on diet (AUROC, area under receiver operating characteristic curve, Western diet WT *versus* knockout mice: 190 *versus* 122, *P*=0.02). All animals were evaluated (four animals per genotype in chow diet groups and eight animals per genotype in Western diet groups). Left panel: insulin tolerance test at 15 weeks on diet (AUROC 167 *versus* 118, *P*=0.0002). (**b**) Fasting glucose, insulin and HOMA. (**c**) Size and area of pancreatic Langherhans islands. (**d**) Representative H&E sections (200x field) from pancreas. Scale bar, 100 μm. (**e**) Total cholesterol, triglycerides and nonesterified fatty acids serum levels. The errors reported represent mean±S.E.M. *P<0.05, **P<0.01 chow *versus* Western diet; ^#^*P*<0.05, ^##^*P*<0.01 WT *versus* knockout mice. Interaction, assessed by two-way ANOVA, indicates that the effect of diet was different between genotypes

Western diet resulted in glucose intolerance in WT mice, as evidenced by higher fasting glucose level, higher peak glucose after challenge and delayed return to baseline. Also, WT mice on Western diet had a blunted response to insulin, indicative of insulin resistance. However, Western diet-fed caspase-2-deficient mice remained glucose tolerant and insulin sensitive (Figure 2a). WT mice fed the Western diet developed overt T2DM, with fasting glucose levels of 200 mg/dl, despite increased insulin levels, and HOMA (homeostatic model assessment)-insulin resistance higher than 15, whereas caspase-2-deficient mice maintained normal glucose metabolism (Figure 2b). As expected,^22, 23^ Western diet induced hyperplasia of pancreatic Langerhans islands in WT mice, but this expansion was significantly blunted in caspase-2-deficient mice (Figure 2c and d). T2DM develops when peripheral insulin resistance surpasses the ability of the pancreas to increase insulin production, resulting in overwhelming stress to insulin-producing *β*-cells and poor insulin responsiveness.^24^ After 16 weeks on Western diet, WT exhibited extreme hyperplasia of Langerhans islands, but had fasting hyperglycemia and impaired late response to glucose challenge, suggesting insufficient insulin secretion and T2DM. In contrast, glucose and insulin dysfunction were largely absent in caspase-2-deficient mice.

### Caspase-2-deficient mice are protected from diet-induced dyslipidemia

In WT mice, Western diet induced mixed dyslipidemia with elevated total cholesterol, triglycerides and nonesterified fatty acids (NEFAs). Caspase-2-deficient mice were protected from dyslipidemia (Figure 2e). The failure of caspase-2 mice to increase circulating NEFA following Western diet may be extremely relevant in the prevention of T2DM, since increased circulating NEFAs are known to decrease glucose transport into muscle cells, and increase both liver gluconeogenesis and pancreatic *β*-cell dysfunction.^25^

### Caspase-2-deficient mice reprogram adipose tissue to cope with energy surplus

**Figure 3:**
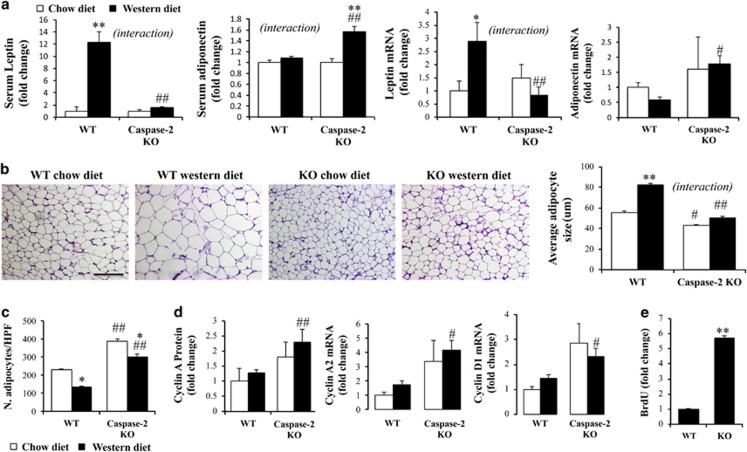
Adipose tissue is fundamentally altered in caspase-2-deficient mice. (**a**) Leptin and adiponectin serum levels and qRT-PCR analysis in adipose tissue from caspase-2 knockout and WT mice fed chow *versus* Western diet (four animals per genotype in chow diet groups and eight animals per genotype in Western diet groups), at sacrifice. (**b**) Representative photos from H&E staining in adipose tissue sections, and average size of adipocytes (100x field). Scale bar, 100 μm. (**c**) Number of adipocytes/HPF. Size and number of adipocytes was assessed in five photos in 100x magnification, from each mouse. (**d**) Cyclin expression (immunoblot, qRT-PCR). The errors reported represent mean±S.E.M, normalized to chow-diet-fed WT mice. *P<0.05, **P<0.01 chow *versus* Western diet; ^#^*P*<0.05, ^##^*P*<0.01 WT *versus* knockout mice. Interaction, assessed by two-way ANOVA, indicates that the effect of diet was different between genotypes. (**e**) Proliferation (BrdU) in adipose tissue mesenchymal stem cells isolated from WT or knockout mice-fed chow diet. The errors reported represent mean±S.E.M from six replicate measurements. The experiment was repeated three times, with similar results

Western diet perturbed adipose tissue homeostasis by deregulating adipokines: increasing adipose tissue expression of leptin and tending to decrease adiponectin expression in WT mice. This deregulation did not occur in caspase-2-deficient mice (Figure 3a). Even in mice-fed chow diet, abdominal adipose tissue from caspase-2-deficient mice was fundamentally different from that of WT mice, with an increased number of small adipocytes (Figure 3b and c). On Western diet, adipocytes in WT mice markedly increased their size, but remained small in caspase-2-deficient mice (Figure 3b). The difference in adipocytes size is highly relevant, since it has been shown that there is an adipocyte size threshold after which the risk of T2DM increases exponentially.^10^ Consistent with the increased number of smaller cells in caspase-2 knockout mice, these cells also had increased expression of cyclins, suggesting increased proliferative activity in the adipose cells of caspase-2-deficient mice (Figure 3d). Isolated adipose stem cells from caspase-2-deficient mice showed increased proliferation compared with WT cells (Figure 3e). The adipose tissue from caspase-2-deficient mice fed Western diet was also protected from fibrosis, as assessed by qRT-PCR analysis for transforming growth factor-*β*, collagen-1*α* and collagen-6*α* (Supplementary Figure 2).

**Figure 4:**
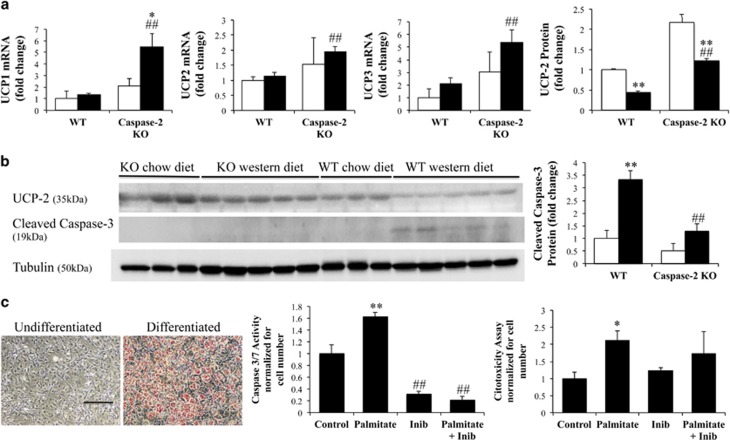
Adipose tissue from caspase-2-deficient mice is resistant to energy surplus induced apoptosis. (**a**) Adipose tissue UCP (qRT-PCR, immunoblot). (**b**) Cleaved caspase-3 expression in adipose tissue (immunoblot). All animals were evaluated (four animals per genotype in chow diet groups and eight animals per genotype in Western diet groups), at sacrifice. The errors reported represent mean±S.E.M., normalized to chow-diet-fed WT mice. *P<0.05, **P<0.01 chow *versus* Western diet; ^#^*P*<0.05, ^##^*P*<0.01 WT *versus* knockout mice. (**c**) Right panel: oil red staining demonstrating 3T3-L1 cell differentiation into adipocytes. Scale bar, 500 μm. Middle panel: caspase-3/7 activity (measured by luminescence, normalized to number of viable cells assessed by fluorescence, according to manufacturer instructions) in adipocytes treated with palmitate (1 mM)±caspase-2 inhibitor (20 uM). Left panel: cytotoxicity assay in the same wells as apoptosis assay. *P<0.05 and **P<0.01 palmitate *versus* control treated cells; ^##^*P*<0.01 cells treated with caspase-2 inhibitor *versus* no treatment

Caspase-2-deficient mice cope better with energy surplus by decreasing the efficiency of energy obtained by burning fat, through increased expression of mitochondrial uncoupling proteins (UCPs), suggesting browning of the adipose tissue (Figure 4a and b; Supplementary Figure 3). UCPs decrease the proton gradient across the mitochondrial inner membrane, decreasing the generation of ATP from burning glucose and fatty acids. Browning of white adipose tissue (that is, genetic or pharmacological increase in UCP function), is protective against the development of obesity-related T2DM.^26, 27^ Moreover, we found an increase in expression of the transcriptional co-activator PGC-1 in the adipose tissue of caspase-2-deficient mice on Western diet, as compared with WT mice, suggesting an increase in mitochondrial biogenesis (Supplementary Figure 3).

Caspase-2 expression decreases as 3T3-L1 pre-adipocytes become adipocytes during culture-induced differentiation.^28^ Relative to WT mice, expression of crucial genes that regulate adipocyte metabolism and differentiation were increased in the adipose tissue of capase-2 deficient mice, including lipoprotein lipase, peroxisome proliferator activator receptor-γ (PPAR-γ); sterol regulatory element-binding protein-1 (SREBP-1), CCAAT-enhancer-binding protein-*α*, (CEBP-*α*) and liver X receptor-*α* (LXR-*α*) (Supplementary Figure 4). Further, adipocytes of caspase-2-deficient mice appeared to be more fit than those of WT mice. For example, cleaved caspase-3, a marker of apoptosis, increased in adipose depots in WT mice, but not in caspase-2-deficient mice, when challenged by chronic exposure to energy-dense Western diets (Figure 4b). Accumulation of pro-apoptotic fatty acids, such as palmitate, typically induces expression of caspase-2 in WT cells. To more directly evaluate the effects of caspase-2 on adipocyte apoptosis, we differentiated 3T3-L1 cells into mature, oil red O-positive adipocytes (Figure 4c, left panel) and treated them with palmitate in the absence or presence of Z-VDVAD-FMK. Z-VDVAD-FMK preferentially binds (and hence, inhibits) caspase-2, and it has been widely used to assess caspase-2 catalytic function. Treatment with the caspase-2 inhibitor blocked palmitate-induced apoptosis in 3T3-L1 adipocytes (Figure 4c, middle panel), supporting the concept that silencing caspase-2 helps mature adipocytes withstand metabolic stress. Inhibition of caspase-2 did not prevent direct palmitate cytotoxicity on 3T3-L1 adipocytes (Figure 4c, right panel).

### Caspase-2-deficient mice are protected from NAFLD

**Figure 5:**
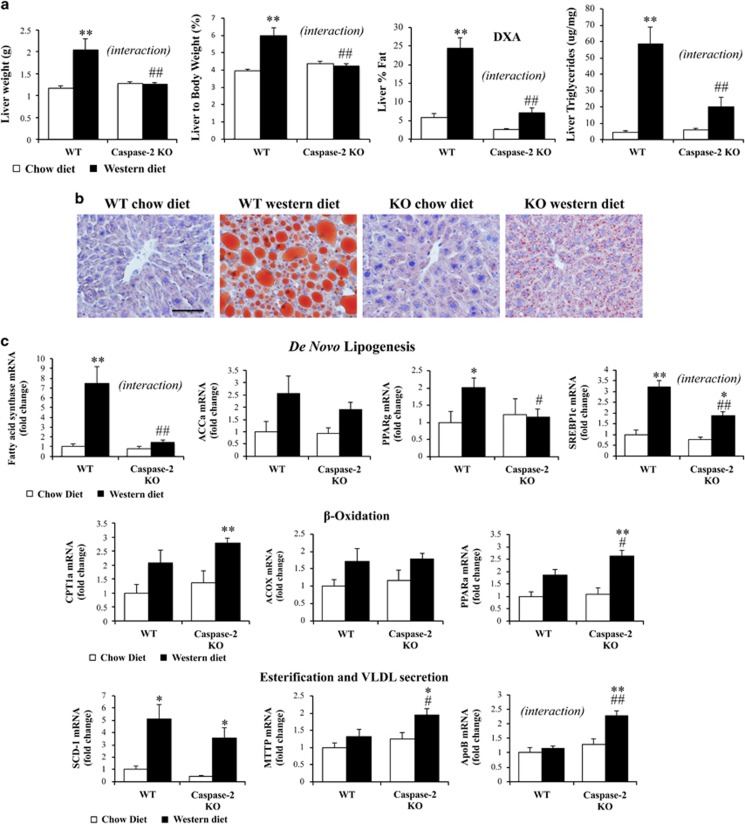
Caspase-2-deficient mice are protected from NAFLD. (**a**) Liver weight, liver-to-body weight ratio, % of liver fat (DXA) and liver triglyceride content. (**b**) Representative photos from oil red staining of liver sections. Scale bar, 100 μm. (**c**) qRT-PCR analysis of liver RNA for genes involved in lipid metabolism, normalized to chow-diet-fed WT mice. All animals were evaluated (four animals per genotype in chow diet groups and eight animals per genotype in Western diet groups). The errors reported represent mean±S.E.M. *P<0.05, **P<0.01 chow *versus* Western diet; ^#^*P*<0.05, ^##^*P*<0.01 WT *versus* knockout mice. Interaction, assessed by two-way ANOVA, indicates that the effect of diet was different between genotypes

NAFLD has been proposed as an additional component of the metabolic syndrome.^29^ Western diet induced massive hepatomegaly in WT mice, with an almost twofold increase in liver mass, and 50% increase in liver-to-body weight ratio. However, caspase-2-deficient mice maintained normal liver weight and liver-to-body weight ratio on Western diet (Figure 5a). Hepatomegaly in WT mice was due to a 5–6-fold increase in liver fat content (as assessed by DXA and triglyceride measurement), whereas caspase-2-deficient mice had strikingly lower hepatic fat accumulation and were protected from Western diet-induced hepatomegaly (Figure 5a and b). Hepatic gene expression analysis showed that caspase-2-deficient mice modulated lipid metabolism towards less fat accumulation, and higher consumption/export of fat. As compared to WT mice, Western diet-fed caspase-2-deficient mice had lower expression of enzymes of *de novo* lipogenesis, and increased expression of enzymes in fatty acid *β*-oxidation, esterification and very-low-density lipoproteins secretion (Figure 5c).

### Caspase-2-deficient mice are protected from liver injury induced by Western diet

**Figure 6:**
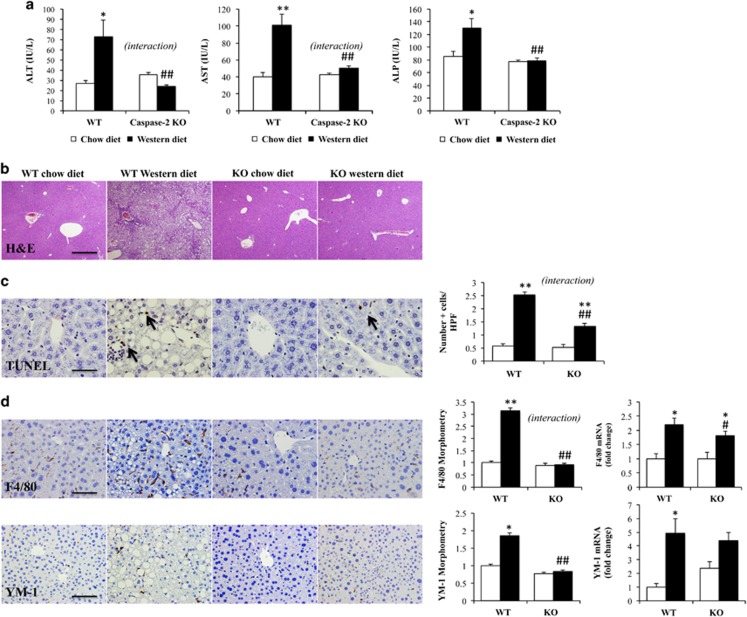
Caspase-2-deficient mice are protected from liver injury induced by Western diet Aminotransferases (ALT, AST) and alkaline phosphatase (ALP) serum levels (**a**) in WT and caspase-2-deficient mice-fed chow (*n*=4/genotype) or Western diet (*n*=8/genotype) for 16 weeks. Representative liver sections stained with H&E (200x field) (**b**), TUNEL with quantification of number of positive cells (**c**), inflammatory markers F4/80 and YM-1 with morphometric quantification and qRT-PCR analysis in whole liver (**d**). Scale bar, 100 μm. The errors reported represent mean±S.E.M., normalized to expression in chow-diet-fed mice. *P<0.05, **P<0.01 chow *versus* Western diet. ^#^*P*<0.05, ^##^*P*<0.01 WT *versus* caspase-2-deficient mice. Interaction, assessed by two-way ANOVA, indicates that the effect of diet was different between genotypes

Previously we showed that caspase-2 expression is markedly upregulated in human nonalcoholic steatohepatitis (NASH), correlating with fibrosis severity in patients.^20^ In the mouse MCD diet model of steatohepatitis, caspase-2 was not only upregulated, but had a crucial role in liver injury and fibrogenesis.^20^ Here we report that there is also a tremendous upregulation of caspase-2 expression in the Western diet mouse model of NASH (Supplementary Figure 5). Western diet induced a 3–4-fold increase in serum levels of aminotransferases and alkaline phosphatase (markers of liver injury) in WT mice, but liver enzyme levels remained in the normal range in caspase-2-deficient mice that were fed Western diet (Figure 6a). We also found less liver injury in caspase-2-deficient mice as assessed by H&E staining (Figure 6b; Supplementary Figure 6), TUNEL staining (Figure 6c) and immunohistochemistry, as well as mRNA liver expression, for markers of classical (F4/80) or alternative (YM-1) macrophage activation (Figure 6d).

### Caspase-2-deficient mice are protected from fibroductular reaction induced by Western diet

**Figure 7:**
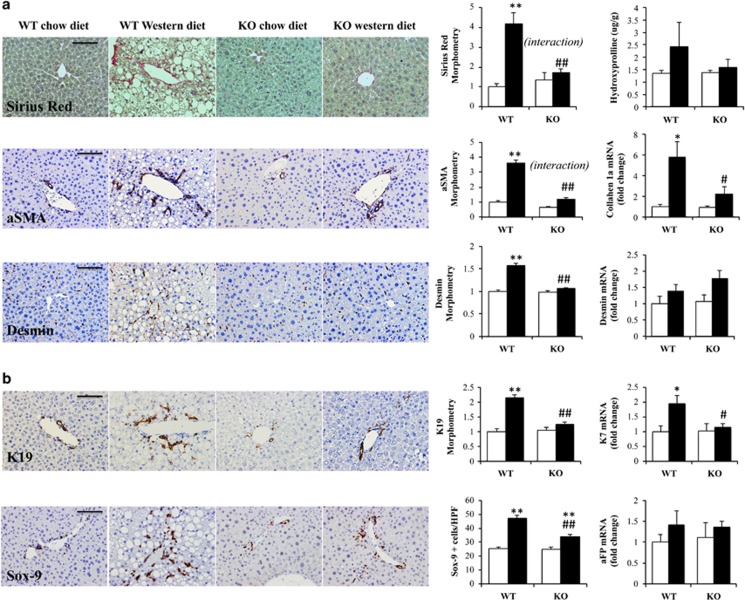
Caspase-2-deficient mice are protected from fibroductular reaction induced by Western diet (**a**). Fibrosis evaluation in the same mice as Figure 2: representative liver sections for Sirius Red staining with morphometric quantification (upper left panels), and liver hydroxyproline quantification (upper right panel). Representative liver sections for immunohistochemistry for stellate cells markers (*α*SMA, desmin) with morphometric quantification (lower left panels) and qRT-PCR in whole liver for collagen-1a and desmin gene expression (right lower panels). (**b**) Ductular reaction evaluated with immunohistochemistry for K19 and Sox-9 with morphometry (left panels), qRT-PCR for K7 and alpha-fetoprotein (aFP) (right panels). Scale bar, 100 μm. The errors reported represent mean±S.E.M., normalized for WT chow diet mice and graphed as mean±S.E.M. *P<0.05, **P<0.01 *versus* chow diet; ^#^*P*<0.05, ^##^*P*<0.01 *versus* WT. Interaction, assessed by two-way ANOVA, indicates that the effect of diet was different between genotypes

The most important known factor for accurate prognosis prediction in NASH is liver fibrosis.^30^ Western diet induced more liver fibrosis in WT mice than in caspase-2-deficient mice, as assessed by Sirius Red staining, hydroxyproline content, qRT-PCR for collagen-1*α*1, and immunohistochemistry for the myofibroblast markers, *α*-smooth muscle actin (*α*-SMA) and desmin (Figure 7a). These results corroborate our previous study of MCD diet-induced steatohepatitis, which also showed that inhibiting caspase-2 provided protection from fibrosis in that model of NASH.^20^ Fibrosis progression in NASH directly correlates with accumulation of reactive appearing bile ductules and inflammatory cells (the ductular reaction).^31^ Compared with WT mice fed Western diet, caspase-2-deficient mice were also protected from diet-induced ductular reaction (Figure 7b).

## Discussion

In a mouse model of diet-induced metabolic syndrome, caspase-2 deficiency protects from the development of key aspects of the metabolic syndrome, including central obesity, dyslipidemia, T2DM and NAFLD. We used a model that mimics a Western diet, with 45% total fat enriched in saturated fats, supplemented with 0.2% cholesterol and with high-fructose corn syrup equivalent to soft drinks. We fed Western diet to WT and caspase-2-deficient mice for 16 weeks, and despite similar food intake, caspase-2-deficient mice gained less weight and fat mass, were more glucose tolerant and insulin sensitive, did not develop dyslipidemia, and were protected from NAFLD. Those effects are associated with a different adipose tissue physiology that could cope better with energy surplus.

The involvement of a protein known to regulate cell death and the cell cycle in regulating whole-body metabolism is non-intuitive. Natural selection optimized mechanisms to cope with food deprivation. When presented with energy surplus, these adaptive mechanisms become deleterious and lead to the development of the metabolic syndrome. Caspase-2, the most evolutionarily conserved caspase,^13^ mediates programmed cell death in models of food deprivation through a mechanism requiring accumulation of toxic fatty acids.^19^ Interestingly, caspase-2 has two isoforms, caspase-2_L_ (long isoform) that induces cell death and is expressed in most tissues, and caspase-2_S_ (short isoform) that antagonizes cell death, and is expressed mainly in brain, heart and skeletal muscle.^32^ This suggests an ancient role for caspase-2 in preserving the most essential organs by sacrificing less critical tissues in order to survive prolonged fasting. However, in modern times, energy surplus can also lead to accumulation of toxic lipids, leading to maladaptive caspase-2 activation.

We propose the following paradigm: Western diet leads to accumulation of toxic lipids in adipocytes. Lipotoxic adipocytes activate caspase-2, inducing cell stress and death, leading to the release of toxic mediators (e.g., adipokines), decreased uptake of NEFAs from blood, and release of NEFAs from dying cells. The net result is an increase in circulating NEFAs and perturbed adipokine secretion, promoting the metabolic syndrome. Supporting this hypothesis, caspase-2-deficient mice fed a Western diet showed some evidence of reduced activation of apoptosis in abdominal adipose tissue, normal adipokine secretion profile, decreased levels of circulating NEFAs, and protection from the development of insulin resistance/T2DM and the metabolic syndrome. These new findings reveal the relevance of increased caspase-2 in adipose tissue of rats fed a high-fat diet,^18^ and complement an earlier report showing that caspase-2-deficient mice resist age-induced glucose intolerance.^16^ Our findings are in concordance with previous work from other labs showing an increase in adipocyte apoptosis both in mouse models of diet-induced obesity and obese humans.^33^ Adipocyte apoptosis correlates with insulin resistance and metabolic disturbances in obesity.^34^ Moreover, adipocyte apoptosis likely plays a causal role in both processes because deletion of the pro-apoptotic factor, Bid, prevented adipose tissue inflammation, systemic insulin resistance, and hepatic steatosis in mice with diet-induced obesity.^33^ The mechanisms involved are being uncovered. For example, there is evidence that apoptosis-effector caspases cleave PPAR-*γ* and GLUT-4, leading to decreased lipogenic gene transcription and impaired glucose uptake, effects that promote insulin resistance.^35^ In aggregate, these data suggest a unifying hypothesis whereby adipocyte apoptosis both leads to a massive decrease in adipose tissue capacity for fat storage by limiting the expansibility of the adipose tissue and induces an insulin resistant and pro-inflammatory state that promotes the metabolic syndrome. As a key initiator of adipocyte apoptosis, caspase-2 activation is a critical driver for this state of ‘relative lipodystrophy' that limits the adaptive response to energy surplus.

We identified several mechanisms in caspase-2-deficient mice that promote adipocyte homeostasis in response to energy surplus. Abdominal adipose tissue from caspase-2-deficient mice is fundamentally different from that of WT mice: adipose stem cells are more proliferative and hence, caspase-2-deficient mice have a larger number of small adipocytes, even when fed chow diet. Fat mass expansion by hyperplasia (increased cell number) is considered a healthier mechanism to compensate for energy surplus than expanding fat mass via adipocyte hypertrophy (increased cell size).^36^ Indeed, in twin pairs discordant for obesity, the prevalence of insulin resistance, metabolic syndrome and NAFLD in obese twins with hyperplastic adipose tissue is similar to that of their lean siblings, whereas obese twins with hypoplastic adipose tissue have higher rates of these conditions than their lean counterparts.^37^ In other studies, adipocyte size also had a strong positive correlation with risk for developing T2DM, cardiovascular disease and NAFLD.^38, 39^ Several putative mechanisms for insulin resistance have been demonstrated in enlarged adipocytes including impaired insulin signaling due to decreased concentration of cholesterol in the cellular membrane,^40^ cytoskeleton disassembly,^41^ and excessive local accumulation of NEFAs.^42^ Fat depots with hypertrophic adipocytes also exhibit oxidative stress,^43^ ER stress,^44^ hypoxia,^45^ inflammation^46^ and fibrogenesis.^47^ These cellular stresses can culminate in adipocyte death, decreasing adipose tissue storage capacity during energy surplus, with the resultant relative lipodystrophy promoting an inflammatory state that characterizes human obesity.

Our study is the first to report increased proliferative capacity in untransformed somatic cells of adult caspase-2-deficient mice. Embryonic fibroblasts from caspase-2-deficient mice were reported to have a higher growth rate after oncogenic transformation than comparable cells from WT mice.^48^ In addition, female caspase-2-deficient-mice are known to have more primordial follicles than WT mice, although this results from reduced apoptosis, rather than increased cell division.^49^ Here we show not only that untransformed adipose stem cells are more proliferative *in vitro*, but also that chow-fed caspase-2-deficient mice have more adipocytes in abdominal adipose tissue *in vivo* than WT mice, and this difference persists after challenge with Western diet. The mechanism by which caspase-2 depletion confers a proliferative advantage is not yet fully understood. Caspase-2 is likely to inhibit replication because its activity is typically repressed during mitosis by a mechanism involving cyclin B1-mediated phosphorylation of caspase-2.^50^ Caspase-2 has been proposed to regulate cell cycle checkpoints through regulation of p53;^51^ p53 also regulates caspase-2 by controlling transcription of p53-induced protein with a death domain (PIDD), a scaffold protein required for caspase-2 activation. On the other hand, caspase-2 negatively regulates the NF*k*B survival and proliferative pathways through cleavage and degradation of receptor-interacting serine/threonine protein kinase 1 (RIPK1)^52^ and competition for assembly with PIDD.^13^

Our data also show, for the first time, that white adipocytes from caspase-2-deficient mice have evidence of browning, as indicated by UCP induction to more effectively cope with energy excess. Caspase-2 can also regulate metabolism through other mechanisms that are not fully understood. For instance, it has been suggested that caspase-2 regulates expression of FoxO1 and FoxO3a, important players in insulin signaling.^53^ Metabolic reprogramming of adipose tissue can also help explain the phenotype of caspase-2-deficient mice. We found increased expression of adipocyte differentiation markers, PPAR-γ, CEBP-*α* and SREBP-1c in caspase-2-deficient mice. These genes control adipocyte triglyceride storage capacity, and are expressed at high levels in adipose tissues of metabolically healthy obese individuals.^54^ In addition, PPAR-γ is a crucial regulator of gene networks involved in glucose homeostasis, controlling adiponectin expression.^55^ LXR-*α* expression was also increased in caspase-2-deficient adipose tissue. LXR-*α* promotes lipolysis and fatty acid stimulated oxygen consumption, and associates with decreased adipocyte size.^56^ The net outcome of these various responses to caspase-2 deletion is adipose depots with a larger number of adipocytes that are able to consume more energy. Hence, caspase-2-deficient mice do not develop stressful enlargement of adipocytes when challenged with an energy-dense Western diet. This is important as increased size/volume of adipocytes correlates with the development of T2DM.^10^ These beneficial effects of caspase-2 deletion of adipose tissue metabolism are likely to contribute to the profound protection from hepatic steatosis in caspase-2-deficient mice fed Western diet. Indeed, caspase-2-deficient mice were not protected from hepatic steatosis when fed MCD diet,^20^ a model in which hepatic steatosis is a consequence of reduced hepatic lipid export, rather than increased import of adipose-derived NEFAs. Additionally, the livers from caspase-2-deficient mice coped better with Western diet-related energy surplus by reprogramming hepatic metabolism, decreasing *de novo* hepatic lipogenesis, increasing hepatic fatty acid oxidation, and increasing fat export from the liver as lipoproteins. As in our prior study of MCD diet-fed mice,^29^ caspase-2 depletion protected Western diet-fed mice from liver injury, inflammation, and fibrosis. Thus, modulating caspase-2 activity is an appealing therapeutic approach for obesity and related metabolic syndrome pathology, including NAFLD.

Capase-2 inhibition appears to be safe in mice. Caspase-2-deficient mice have a near normal phenotype and do not develop excessive tumors with aging, despite the fact that caspase-2 is an inhibitor of apoptosis and as such, might have some tumor suppressing functions. Specific inhibition of caspase-2 is also predicted to be safer than compromising the function of multiple caspases by treatment with pan-caspase inhibitors. Nevertheless, some reports have suggested that caspase-2 deficiency might lead to premature aging by reducing the clearance of cells damaged by oxidative stress.^16, 57^ Our studies, on the other hand, demonstrated a decrease in the level of liver oxidative stress caused by Western diet. This result is consistent with evidence that caspase-2 deficiency reduced diet-induced lipid accumulation, because ectopic fat accumulation is a major driver of oxidative stress.

Our work raises several questions that must be further addressed. The biochemical mechanism by which caspase-2 deficiency protects from metabolic dysregulation remains unidentified. This must be clarified in order to design an appropriately targeted intervention that abrogates the deleterious effects of caspase-2 during energy excess. Current understanding of caspase-2 biochemistry suggests that caspase-2 might drive metabolic stress either via its proteolytic-catalytic activity (which is pro-apoptotic) or its scaffolding functions (which mediate the molecule's actions on proliferation and gene expression). Further, because no specific inhibitor of caspase-2 has been developed yet, it has not been possible to test the ability of such an agent to recapitulate the effects of caspase-2 gene deletion. Nevertheless, the current study provides compelling novel evidence that caspase-2 is a potential target to correct obesity and its associated comorbidities: metabolic syndrome, insulin resistance/T2DM and NAFLD. These diseases are the major killers of the Western world, and approaches to treat or prevent them will have tremendous implications for overall human health.

## Materials and Methods

### Animal studies

Male caspase-2-deficient and congenic WT C57Bl/6J mice were obtained from Jackson Laboratory (Bar Harbor, ME, USA). Bergeron *et al.* inactivated the caspase-2 gene by homologous recombination in 129S4/SvJae-derived J1 embryonic stem cells, and the founder mice were backcrossed to C57BL/6J inbred mice for 10 generations by Bergeron *et al.* before being donated to the Jax Lab, where they were C57Bl/6J-backcrossed for at least one more generation, resulting in genetic uniformity. Mice were fed either chow diet (Picolab Rodent diet 20, #5053; *n*=4 mice per genotype) or Western diet (TD.120330 22% HVO+0.2% cholesterol diet, Teklad Research, supplemented with fructose and glucose in the drinking water; *n*=8 mice per genotype) for 16 weeks, beginning at 4 weeks of age. Diet specifications are in Supplementary Tables 1 and 2.

During the last week of diet, glucose and insulin tolerance tests were performed. Glucose tolerance test was performed after 12 h of fasting, by IP injection of glucose (2 g/kg). Insulin tolerance test was performed after 5 h of fasting, with IP injection of 0.6 units/kg of human regular insulin. Glucose levels were measured at sequential time points by tail vein sampling using a glucometer.

In mice, body mass index was calculated as the body weight (g)/(crown-rump length (mm))^2^, as previously described.^58^

Animal care and procedures were approved by the Duke University Institutional Animal Care and fulfilled National Institutes for Health and Duke University IACUC requirements for humane animal care.

### Histopathological, serum and tissue analysis

Formalin-fixed, paraffin-embedded liver, pancreas and epididymal adipose tissue biopsies were cut into 5 *μ*m serial sections for H&E and Oil red staining. Immunohistochemistry was performed as previously described,^20^ with the primary antibodies specified in Supplementary Table 3.

Insulin was measured with Ultrasensitive Mouse Insulin ELISA kit (Crystal Chem Inc, Cayman Chemical Company, Ann Arbor, MI, USA, #90080), after 12 h fasting. Lipids were measured with Triglyceride Colorimetric Assay kit (Cayman Chemical Company, #10010303), Free Fatty Acid Quantification Kit, (Abcam, Cambridge, MA, USA, ab65341) and Cholesterol Quantification kit (Abcam, ab65359); serum leptin and adiponectin were determined with Abcam mouse ELISA kits, ab100718 and ab108785, respectively.

### Molecular studies

#### mRNA quantification by RT-PCR

Total RNA was extracted from tissue using TRIzol (Invitrogen, Grand Island, NY, USA). RNA was reverse transcribed into cDNA templates using random primer and SuperScript RNAse HReverse Transcriptase (Invitrogen). Semiquantitative qRT-PCR was performed using iQ-SYBR Green Supermix (Bio-Rad, Hercules, CA, USA) on a StepOne Plus Real-Time PCR Platform (ABI/Life Technologies, Carlsbad, CA, USA), as previously described.^59^ For primers, see Supplementary Table 4.

#### Western blotting

Total proteins were extracted from liver and adipose tissue using RIPA buffer (Sigma, St. Louis, MO, USA). Equal amounts of protein were separated by electrophoresis on 4–20% Criterion gels (Bio-Rad), transferred onto polyvinylidene difluoride membranes, and incubated with the primary antibodies specified in Supplementary Table 5.

### Cell isolation and culture

Mouse adipose tissue stem cells were isolated from caspase-2-deficient and WT mice as previously described.^60^

3T3-L1 cells were differentiated into adipocytes,^61^ and differentiation confirmed with Oil red staining. Differentiated adipocytes were treated with 1 mM palmitate without or with 20 μM caspase-2 inhibitor Z-VDVAD-FMK (R&D Systems, Minneapolis, MN, USA) for 48 h.

Apoptosis was measured using ApoTox-Glo Triplex Assay (Promega, San Luis Obispo, CA, USA) and proliferation using BrdU assay (Cell Signaling, Danvers, MA, USA).

### Statistics

Results are expressed as mean±S.E.M. Significance was established using two-way ANOVA, with *P*<0.05 considered significant.

## Abbreviations

AUROC: area under receiver operating characteristic curve
CEBP-*α*: CCAAT-enhancer-binding protein-*α*
DXA: dual-energy X-ray absorptiometry
HOMA: homeostatic model assessment
LXR-*α*: liver X receptor-*α*
MCD: methionine–choline deficient
NAFLD: nonalcoholic fatty liver disease
NASH: nonalcoholic steatohepatitis
NEFA: nonesterified fatty acids
PIDD: p53-induced protein with a death domain
PPAR-*γ*: peroxisome proliferator activator receptor-*γ*
*α*-SMA: α-smooth muscle actin
SREBP-1: sterol regulatory element-binding protein-1
T2DM: type 2 diabetes mellitus
UCP: uncoupling proteins
WT: wild type
