# Supplementary material for: Caspase-2 promotes obesity, the metabolic syndrome and nonalcoholic fatty liver disease
Source: Cell Death Dis. 2016 Feb 18;7(2):e2096–. doi: 10.1038/cddis.2016.19 (PMC5399190; doi:10.1038/cddis.2016.19)
Supplement: Supplementary Information [file cddis201619x1.docx]

**Supplemental Material For the Manuscript**

**Ancestral starvation-activated caspase promotes obesity, the metabolic syndrome and nonalcoholic fatty liver disease**

Mariana Verdelho Machado^1,2^, Gregory A. Michelotti^1^, Mark Jewell^1^, Guanhua Xie^1^, Thiago de Almeida Pereira^1^, Richard T. Premont^1^, Anna Mae Diehl^1^

**SUPPLEMENTAL TABLES**

**Supplemental Table 1** – Characteristics of the diets

|  | Chow diet | Western diet |
| --- | --- | --- |
| Reference | Picolab© Rodent diet 20, #5053 | TD.120330, 22% HVO + 0.2% cholesterol diet, Teklad Research |
| Kcal/g of diet | 4.7 | 4.6 |
| % Fat as calories | 13 | 45.3 |
| % CH as calories | 62 | 37 |
| % Proteins as calories | 24 | 17.7 |

**Supplemental Table 2.** Composition of the experimental diet

|  | Western diet^*^ |
| --- | --- |
| Reference | TD.120330, 22% HVO + 0.2% cholesterol diet, Teklad Research |
| Formula (g/Kg) | Casein (230.0)  DL-Methionine (3.4)  Sucrose, fine ground (211.7098)  Corn Starch (80.0)  Maltodextrin (140.0)  Vegetable Shortening, hydrogenated (Primex) (220.0)  Soybean Oil (10.0)  Cholesterol (2.0)  Cellulose (50.0)  Mineral Mix, AIN-93G-MX (94046) (46.0)  Calcium Phosphate, dibasic (3.3)  Niacin (0.042)  Calcium Pantothenate (0.0224)  Pyridoxine HCl (0.0098)  Thiamin HCl (0.0084)  Riboflavin (0.0084)  Folic Acid (0.0028)  Biotin (0.0003)  Vitamin B12 (0.1% in mannitol) (0.035)  Vitamin E, DL-alpha tocopherol acetate (500 IU/g) (0.1)  Vitamin A Palmitate (500,000 IU/g) (0.0112)  Vitamin D3, cholecalciferol (500,000 IU/g) (0.0028)  Vitamin K1, phylloquinone (0.0011)  Choline Bitartrate (3.3)  TBHQ, antioxidant (0.046) |

*This diet is a modification of TD.06303 to add 0.2% cholesterol. We also add 42 g/L glucose and fructose in the drinking water (55% fructose and 45% glucose, w/w).

**Supplemental Table 3** – Primaries Antibodies for Immunohistochemistry

| **Antibody** | **Host** | **Company** | **Catalog #** | **Retrieval** | **Dilution** |
| --- | --- | --- | --- | --- | --- |
| Caspase-2_L_ | Rabbit | Santa Cruz | Sc-626 | Citrate | 1:3000 |
| F4/80 | Rat | AbD Serotec | MCA497GA | Citrate | 1:150 |
| YM-1 | Rabbit | Stem Cell Technologies | #01404 | Citrate | 1:4000 |
| α-SMA | Rabbit | Abcam | Ab32575 | Citrate | 1:400 |
| Desmin | Rabbit | Abcam | Ab15200 | Citrate | 1:400 |
| K19 | Rat | Develpmental Studies Hybridoma Bank | Troma III | Pepsin + EDTA | 1:150 |
| Sox-9 | Rabbit | EMD Millipore | AB5535 | Citrate | 1:4000 |
| 4-Hydroxynonenal | Rabbit | Abcam | Ab46545 | Citrate | 1:250 |

α-SMA, alpha smooth muscle actin; K19, Keratin 19.

**Table 2** – RT-PCR primers for analysis

| *Gene* | *Primer forward* | *Primer reverse* |
| --- | --- | --- |
| S9 | GACTCCGGAACAAACGTGAGGT | CTTCATCTTGCCCTCGTCCA |
| CPT-1a | TCCACCCTGAGGCATCTATT | ATGACCTCCTGGCATTCTCC |
| PPAR-α | AGAGCCCCATCTGTCCTCTC | ACTGGTAGTCTGCAAAACCAAA |
| ACO | ATGCCTTTGTTGTCCCTATC | CCATCTTCAGGTAGCCATTATC |
| SCD-1 | CGTCTGGAGGAACATCATTC | AGCGCTGGTCATGTAGTA |
| MTTP | TCTCACAGTACCCGTTCTT | TCTTCTCCGAGAGACATATCC |
| ApoB | GGACTGTCTGACTTCCATATTC | AAGACTTGCCACCCAAAG |
| FAS | CTGCGGAAACTTCAGGAAATG | GGTTCGGAATGCTATCCAGG |
| ACC-α | AGGAGGACCGCATTTATCGAC | TGACCGTGGGCACAAAGTT |
| PPAR-γ | AGGCCGAGAAGGAGAAGCTGTTG | TGGCCACCTCTTTGCTCTGCTC |
| SREBP-1c | GCTACCGGTCTTCTATCAATG | GCAAGAAGCGGATGTAGTC |
| Caspase-2 | CAATGCTAACTGTCCAAGTCTA | GGGATTGTGTGTGGTTCTT |
| Leptin | TGACACCAAAACCCTCATCA | CCAGGTCATTGGCTATCTGC |
| Adiponectin | TCTCCAGGAGTGCCATCTCT | GTTGCAAGCTCTCCTGTTCC |
| Cyclin D1 | TAGGCCCTCAGCCTCACT | CCACCCCTGGGATAAAGCAC |
| Cyclin D2 | CCCGACTCCTAAGACCCAT | TTCAGCTTACCCAACACTACCA |
| Cyclin A2 | GGCTGCACCAACAGTAAA | GGGTCAGCATCTATCAAACTC |
| UCP-1 | AGGCTTCCAGTACCATTAGGT | CTGAGTGAGGCAAAGCTGATTT |
| UCP-2 | ATGGTTGGTTTCAAGGCCACA | CGGTATCCAGAGGGAAAGTGAT |
| UCP-3 | CGAATTGGCCTCTACGA | TGTAGGCATCCATAGTCCC |

CPT1a, Carnitine Palmitoyltransferase 1A; PPAR-α, Peroxisome Proliferator-Activated Receptor-α; ACOX, Acyl Coenzyme A Oxidase; SCD-1, Stearoyl-CoA Desaturase; MTTP, Microsomal Triglyceride Transfer Protein; ApoB, Apolipoprotein B; FAS, Fatty Acid Synthase; ACC-α, Acetyl-CoA Carboxylase-α; PPAR-γ, Peroxisome Proliferator-Activated Receptor-γ; SREBP-1c, Sterol Regulatory Element Binding Protein-1c; UCP, uncoupling protein.

**Supplemental Table 5** – Primaries Antibodies for Western blot

| **Antibody** | **Host** | **Company** | **Catalog #** | **Dilution** |
| --- | --- | --- | --- | --- |
| α-Tubulin | Mouse | Abcam | Ab4074 | 1:5000 |
| Cyclin A | Rabbit | Santa Cruz Biotechnology | Sc-596 | 1:500 |
| UCP-2 | Goat | Santa Cruz Biotechnology | Sc-6525 | 1:500 |
| Cleaved Caspase-3 | Rabbit | Cell Signalling | #9661 | 1:500 |

UCP, uncoupling protein.

**SUPPLEMENTAL FIGURES**

**Supplemental Figure 1.**

**Caspase-2 deficient mice are protected from weight gain with Western diet.**

A. Body weight curve in caspase-2 deficient mice and WT mice fed chow diet or Western diet (4 animals per genotype in chow diet groups and 8 animals per genotype in Western diet groups), during 16 weeks of treatment. B. body length from the same mice, at sacrifice. The errors reported represent mean±S.E.M. # P<0.05, ## P<0.01 WT *vs.* knockout mice.

**Supplemental Figure 2**

**Adipose tissue from caspase-2 deficient mice is protected from fibrosis induced by Western diet.**

qRT-PCR analysis of adipose tissue from caspase-2 and WT mice fed chow or Western diet (4 animals per genotype in chow diet groups and 8 animals per genotype in Western diet groups), at sacrifice, for markers of fibrogenesis. The errors reported represent mean±S.E.M., normalized to expression in chow-diet fed mice. * P<0.05, ** P<0.01 chow *versus* Western diet; # P<0.05, ## P<0.01 WT *vs.* knockout mice. Interaction, assessed by 2-way ANOVA, indicates that the effect of diet was different between genotypes.

**Supplemental Figure 3**

**Evaluation of browning of adipose tissue in caspase-2 deficient mice**

A. Representative UCP-1 immunohistochemistry of epididymal tissue isolated from either WT or caspase-2 deficient mice fed either chow diet or western diet (4 animals per genotype in chow diet groups and 8 animals per genotype in Western diet groups, at sacrifice), and respective morphometry. Barr=100 μm. B. qRT-PCR analysis of adipose tissue from the same mice for a marker of mitochondrial biogenesis. The errors reported represent mean±S.E.M., normalized to expression in chow-diet fed mice. * P<0.05, ** P<0.01 chow *versus* Western diet; # P<0.05, ## P<0.01 WT *vs.* knockout mice. Interaction, assessed by 2-way ANOVA, indicates that the effect of diet was different between genotypes.

**Supplemental Figure 4**

**Metabolic profile of adipose tissue from caspase-2 deficient mice.**

qRT-PCR analysis of adipose tissue from caspase-2 and WT fed chow or Western diet (4 animals per genotype in chow diet groups and 8 animals per genotype in Western diet groups, at sacrifice) of major genes in the regulation of metabolism. The errors reported represent mean±S.E.M., normalized to expression in chow-diet fed mice. * P<0.05, ** P<0.01 chow *versus* Western diet; ; # P<0.05, ## P<0.01 WT *vs.* knockout mice. Interaction, assessed by 2-way ANOVA, indicates that the effect of diet was different between genotypes.

**Supplemental Figure 5**

**Low expression of caspase-2 in the healthy liver is highly induced by Western diet.**

A. qRT-PCR analysis of mouse primary hepatocytes, kupffer cells, liver sinusoidal endothelial cells (LSEC), hepatic stellate cells (HSC) and a cholangiocyte cell line (603B cells), for caspase-2 and cell-type specific markers. The errors reported represent mean±S.E.M., for 3 replicates. B. Liver sections from representative WT mice fed chow diet (n=4) or Western diet (n=8), stained for caspase-2 and respective morphometry (left panels), as well as gene expression by qRT-PCR (right panel). Barr=100 μm. C. Immunoblot for caspase-2 from whole liver extracts from the same mice. The errors reported represent mean±S.E.M., normalized to expression in chow-diet fed mice. * P<0.05, ** P<0.01 chow *versus* Western diet.

**Supplemental Figure 6**

**Caspase-2 deficient mice are protected from NAFLD.**

Liver sections from representative WT and caspase-2 deficient mice fed chow diet or Western diet, stained with H&E. Barr=50 μm.
