# Supplementary figures and images for: Caspase-2 promotes obesity, the metabolic syndrome and nonalcoholic fatty liver disease
Source: Cell Death Dis. 2016 Feb 18;7(2):e2096–. doi: 10.1038/cddis.2016.19 (PMC5399190; doi:10.1038/cddis.2016.19)

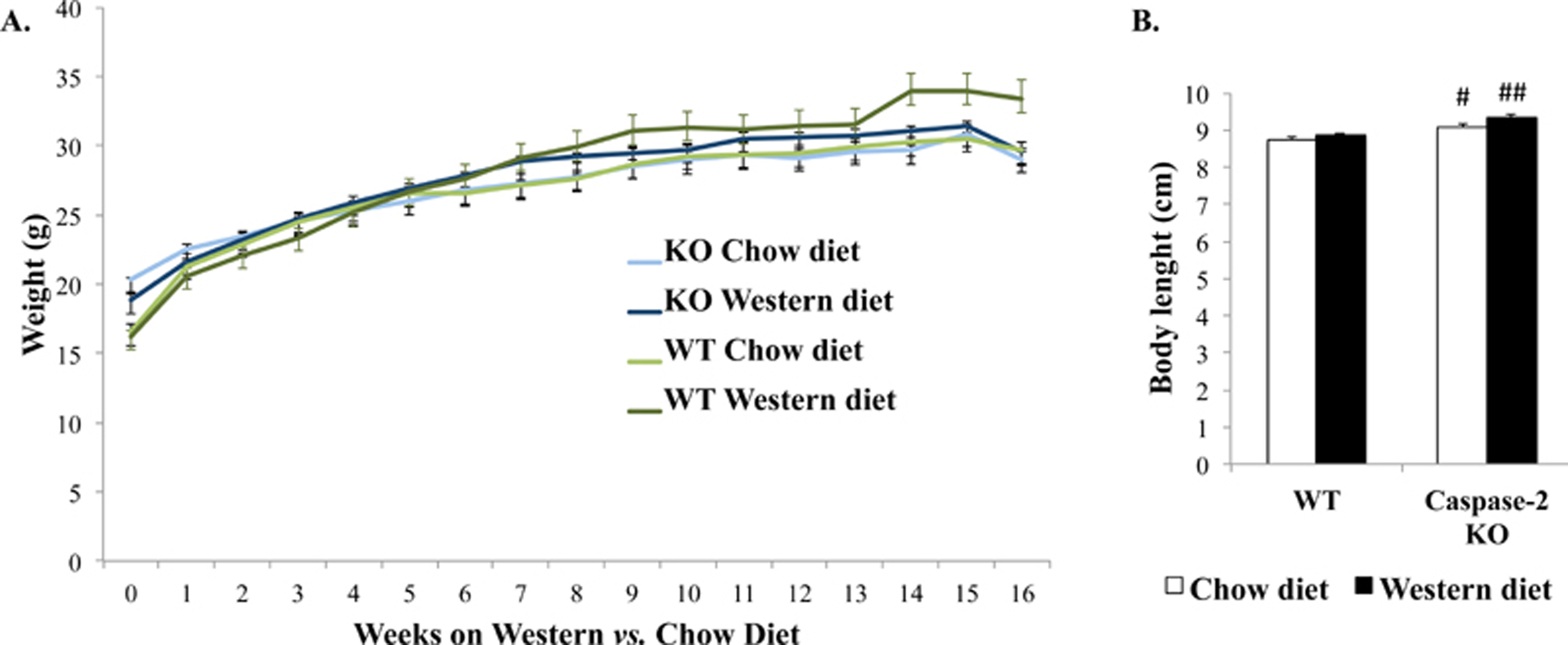

Supplement: Supplementary Figure 1 [file cddis201619x2.tif]

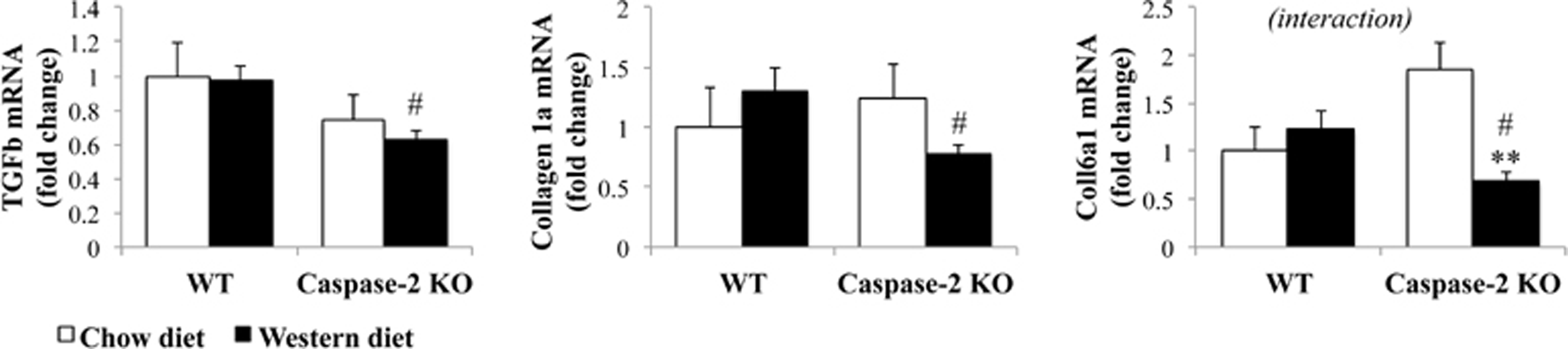

Supplement: Supplementary Figure 2 [file cddis201619x3.tif]

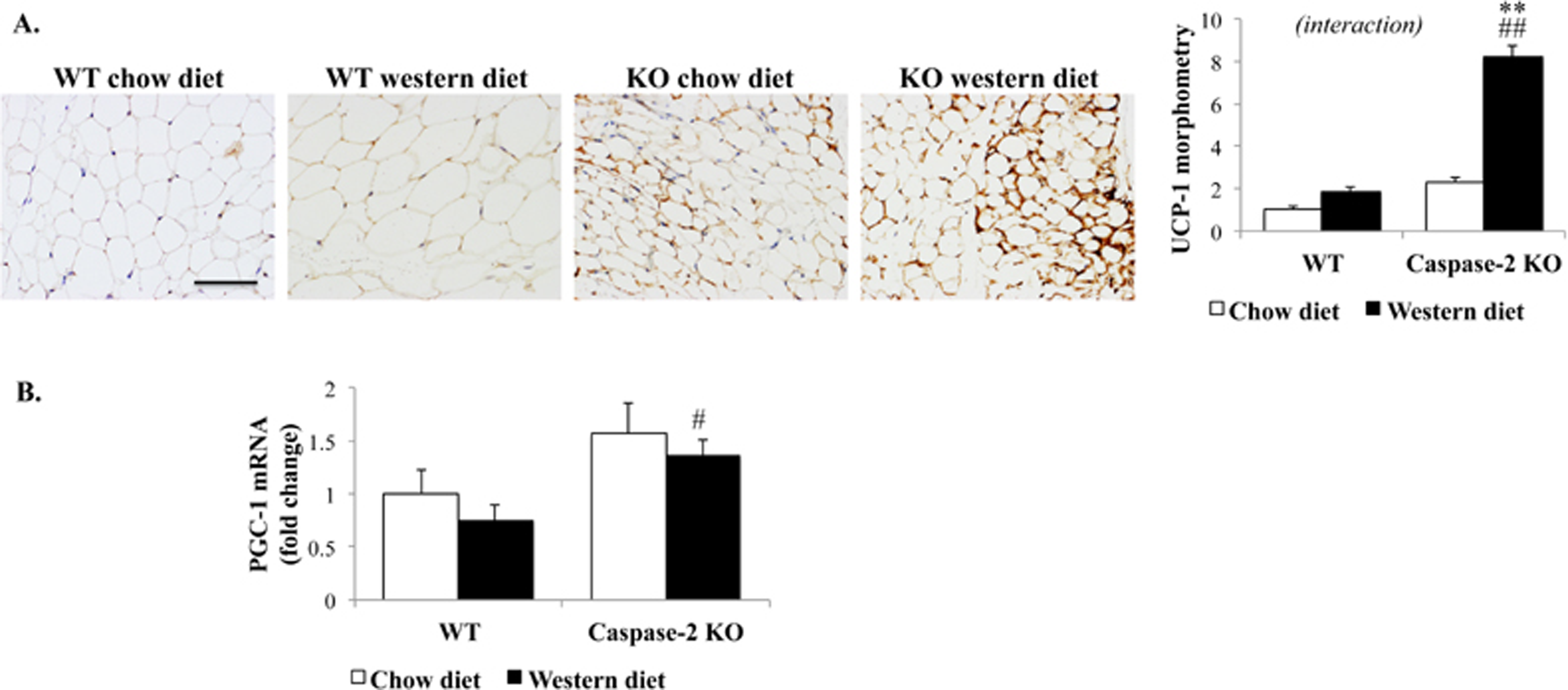

Supplement: Supplementary Figure 3 [file cddis201619x4.tif]

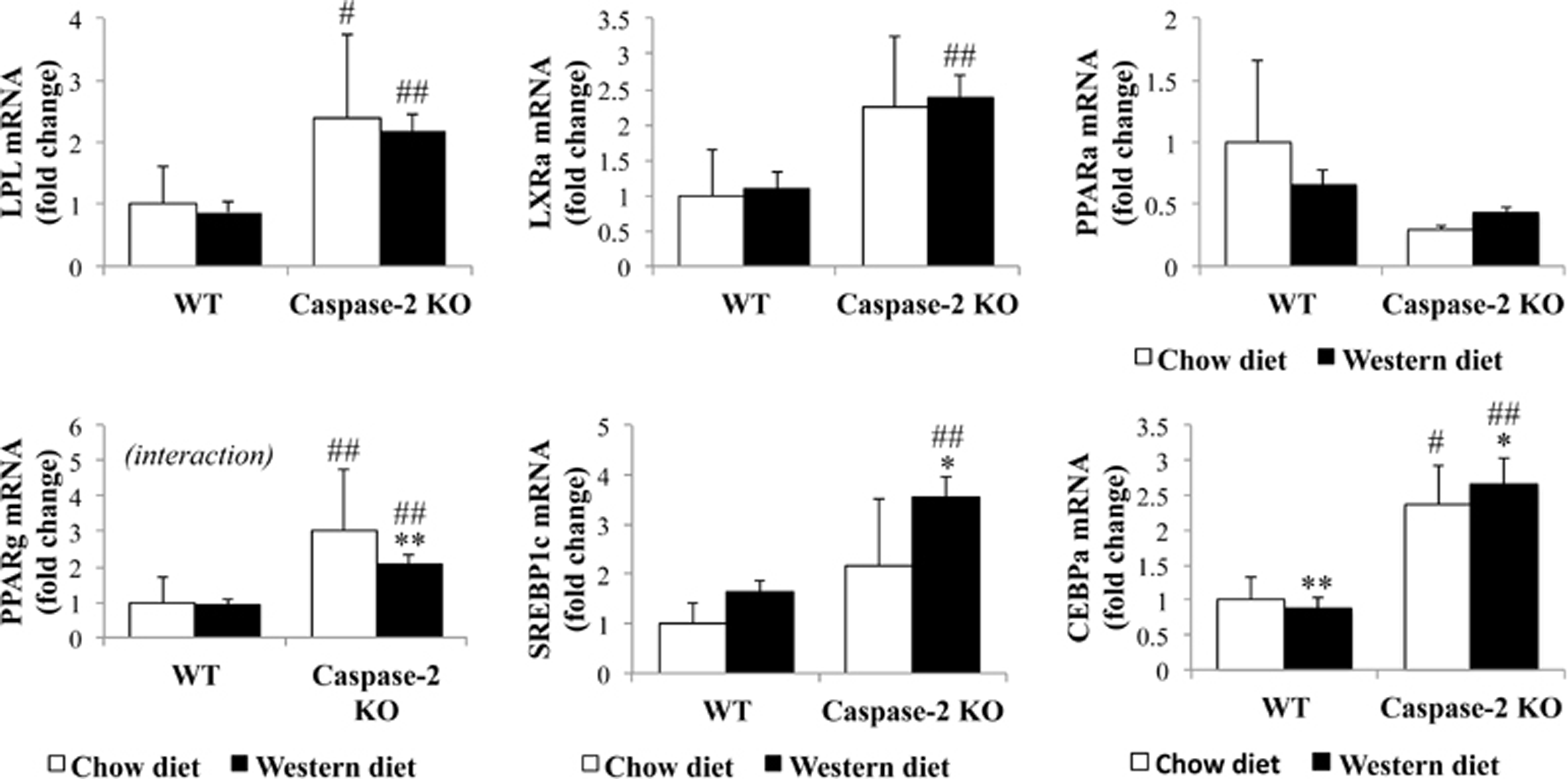

Supplement: Supplementary Figure 4 [file cddis201619x5.tif]

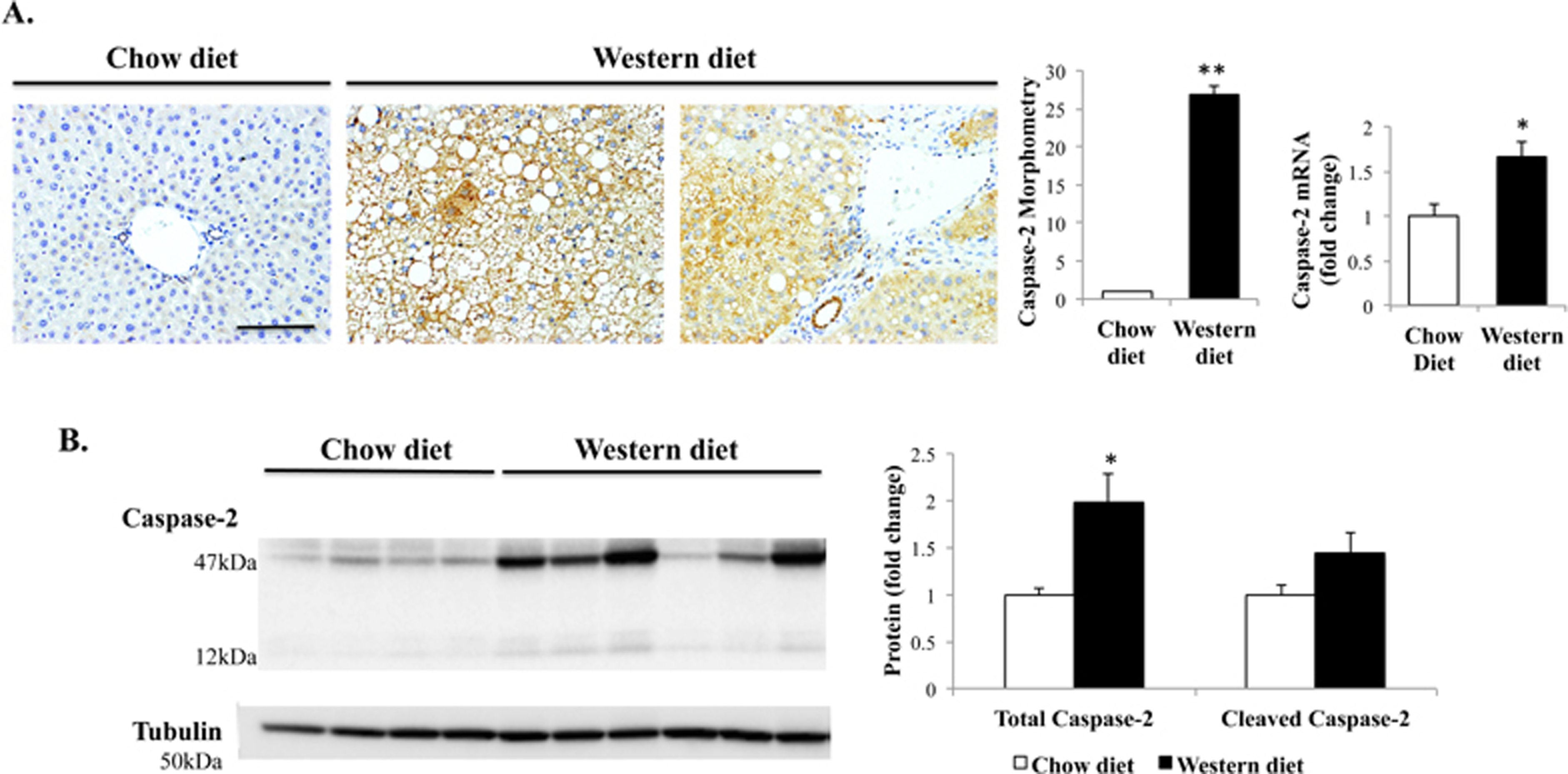

Supplement: Supplementary Figure 5 [file cddis201619x6.tif]

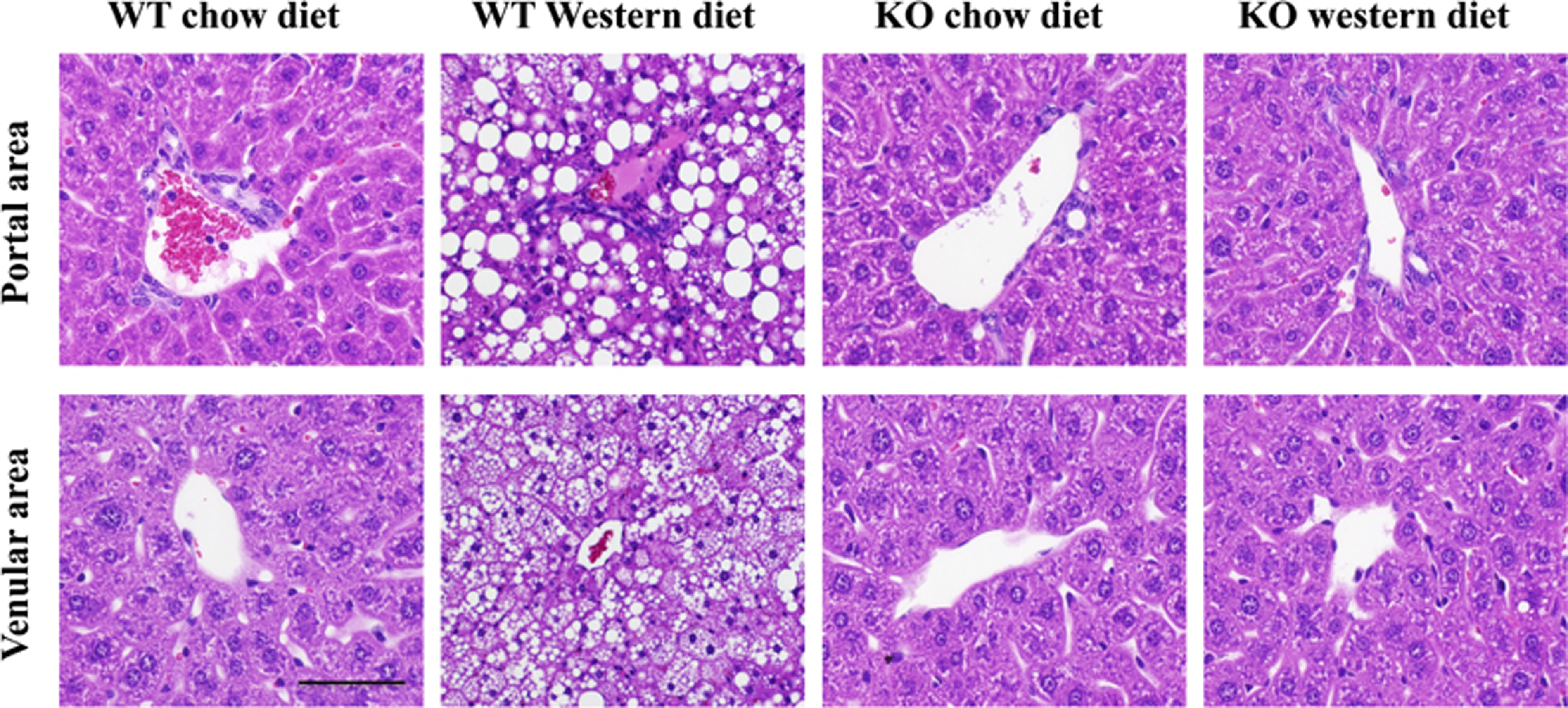

Supplement: Supplementary Figure 6 [file cddis201619x7.tif]
